# Frost Resistance and Pore Structure of Concrete Incorporated with Rubber Aggregates and Nano-SiO_2_

**DOI:** 10.3390/ma14051170

**Published:** 2021-03-02

**Authors:** Jun Fang, Lei Zhao, Jicun Shi

**Affiliations:** 1 School of Civil Engineering and Architecture, Wuhan University of Technology, Wuhan 430070, China; fangjun@whut.edu.cn; 2 School of Civil Engineering and Architecture, Xinxiang University, Xinxiang 453003, China; jicun.shi070@xxu.edu.cn

**Keywords:** concrete, frost-resistant, freeze–thaw, pore structure, rubber, nanosilica

## Abstract

This paper aims to develop frost-resistant concretes, and investigate their pore structures and freeze–thaw damage mechanism. The frost-resistant concrete mixtures are designed by using rubber particles and nano-SiO_2_ to partially replace sands. The chord lengths, specific surface areas, contents and spacing coefficients of the pores in the designed concretes are measured and analyzed. The results show that concrete mixture incorporated with 5% silanized rubber and 3% nanosilica shows good synergetic effect by considering both mass loss and relative dynamic modulus of elasticity (RDME). The freeze–thaw damage degree of the concrete could be reduced by adding high elastic rubber particles, due to filling and constraining pores, and resulting in better uniform pore distribution and smaller pore spacing coefficient. Furthermore, the correlations between frost resistance and pore are analyzed and proposed.

## 1. Introduction

Frost resistance is one of the key parameters of concrete durability, which can be improved by adding air entraining agent into concrete [1]. With the development of science and technology and the protection of the ecological environment, the frost resistance of concrete can be significantly improved by adding waste rubber into concrete [2,3]. At the same time, due to the small particle size and large specific surface area of nano-SiO_2_, adding the same amount of nano-SiO_2_ instead of cement into concrete can give play to its fine particle filling, good pozzolanic and nucleation effects, improving the interface transition zone structure, compactness, and strength, especially the early strength and frost resistance [4].

However, due to the significant difference of physicochemical properties between organic rubber and inorganic cement concrete, the process of combining them is often complex, resulting in poor compressive performance [5]. The addition of silane coupling agent is conducive to the wetting of inorganic materials and the chemical bonding between the carbon functional groups in silane coupling agent and the organic functional groups in polymer. The two materials with different properties can be combined well through chemical bonding, which can increase the bonding strength of the interface layer of the composite, improve the compression and frost resistance of the composite, and reduce the cost.

The freeze–thaw damage of concrete has been a critical scientific topic since the 1940s, and some hypotheses and frost damage theories have been proposed and researched [6]. Some researchers mainly research the relative macro-performance of frost-resistant concrete, and analyze the macroscopic phenomenon by properties in mesoscale and microscale levels [7,8]. Nowadays, the investigation of microstructures attracts a lot of attention in the field of frost-resistant concrete research. The concrete matrix and microstructure are mainly determined by aggregates, cementitious hydration productions and the interfacial transition zone (ITZ, thickness of 10 µm–50 µm). The properties of the aggregates (e.g., density, shape, porosities etc.) have great influence on the concrete’s mechanical strength, as well as the elastic modulus, density and volume stability [9]. The cementitious hydration product including solid phase, moisture and porosity affect shrinkage and creep [10]. The ITZ is usually the weakest part in the concrete, which has a relatively higher porosity and more microcracks, and is enriched by ettringite and Ca(OH)_2_ [11].

In order to improve the frost resistance of concrete, some probable measurements have been investigated, such as using hydrophobic coatings, adding air entraining agents and optimizing the matrix by modified raw materials [12,13,14]. Rubber aggregates have been proved to reduce the degree of freeze–thaw damage [15]. However, the optimal content of rubber, and the weak interfacial bond between the rubber and the matrix have still not been well researched or solved. In the meantime, the rubber aggregates generally tend to reduce the concrete’s mechanical strength [16]. Utilizing nanosilica could efficiently enhance the microstructure and mechanical strength, attributed to their pozzolanic effect, filler effect and nucleation effect [17]. Hence, it is necessary to design frost-resistant concrete combined with rubber aggregates and nanosilica, and analyze its freeze–thaw damage mechanism.

The frost resistance and mechanical properties are dependent on the pore structure, such as pore morphology and pore size distribution [18]. Then, frost resistance and mechanical properties would influence the service life of the concrete structure. However, the pore structure of concrete is very complex and described by many key parameters such as porosity, diameter, chord length, specific surface, spacing coefficient, etc. [19]. Hence, how to analyze the frost resistance by those key pore parameters is still a very important issue, and probable correlations between the pore structure and frost resistance of concrete need to be proposed. This paper intends to investigate frost resistance and pore structure of concretes incorporated with rubber aggregates and nano-SiO_2_.

## 2. Experimental Program

### 2.1. Raw Materials and Mix Proportion

The mixtures are designed including following materials: cement P.C. 32.5R, fly ash, nanosilica, river sand, coarse aggregate, rubber particle, plasticizer and water. The physical and chemical properties of cement are shown in Table 1. The properties of nano-SiO_2_ are listed in Table 2. The particle size of coarse aggregate is from 5 mm to 20 mm, and its specific density is 2.71 g/cm^3^. The rubber particles are produced from recycled waste tires, the average particle size is 140 µm and specific density is 1.11 g/cm^3^. In order to improve the bond between rubber and matrix, both normal and silanized rubber particles, respectively, are utilized and researched in this paper. 

The recipe of the control concrete is shown in Table 3. To research the effects of nano-SiO_2_, rubber and silanized rubber on the performance of frost-resistant concrete, another six mixtures are designed based on the control concrete, as shown in Table 4. The nano-SiO_2_ is added by partially replacing powder (cement and fly ash) by mass. The contents of rubber and silanized rubber particles are calculated by the mass of cement, and partially replace the sand by volume.

### 2.2. Testing Methods

#### 2.2.1. Freeze–Thaw Cycle Test

The freeze–thaw cycle test is conducted according to the Chinese standard GB/T50082-2009 [20]. The cubic samples for freeze–thaw cycle test are made and cured as in Section 2.2.1. After 28 days curing, the samples are immerged in water for about 4 days under temperature of about 20 °C. After that, the concrete samples are put into freeze–thaw set-up. After certain freeze–thaw cycles, the mass loss and relative dynamic modulus of elasticity (RDME) are measured by ultrasonic pulse velocity.

#### 2.2.2. Pore Structure

To analyze the pore structure of concrete after a freeze–thaw cycle test, a commercial pore structure analyzer (Airvoid, Hirek, Beijing, China) is utilized. Each concrete sample is cut and polished into 6 square slices (100 mm × 100 mm × 10 mm). Then, the intersections of concrete slices are scanned by the Airvoid with a testing resolution of 2 µm.

The parameters and characteristics of the pores should have great impact on the mechanical and frost resistance properties of concrete [21]. To comprehensively and completely analyze the key parameters of pores on concrete performance, this paper measures and calculates the average chord length of the pore (*m_l_*), specific surface area of the pore (*a*), the air content (*A*) and the average pore spacing coefficient (*L*).

(1) ml=∑lN

(2) a=4ml

(3) A=100×S1S

(4) L={P4nl,PA≤4.3423A4nl[1.4(PA+1)13+1],PA>4.342

where *l* is pore diameter. *N* is total number of pores. *n_l_* is number of pores per unit volume. *S*_1_ and *S* are the pore area and whole concrete area, respectively. *P* is the volume rate of hardened cement paste without air pores, %.

In order to characterize the propagation mechanism of pore structures under freeze–thaw cycles, the concrete pores can be categorized into four types based on the pore size distribution and testing result [22], as shown in Table 5.

#### 2.2.3. Scanning Electron Microscope (SEM) Test

In this paper, the microstructure characteristics of freeze–thaw damaged concrete samples were observed by FEI QUANTA 250 (FEI, Hillsboro, OR, USA) scanning electron microscope.

## 3. Results Analysis

### 3.1. Microstructure of the Concrete

**Figure 1:**
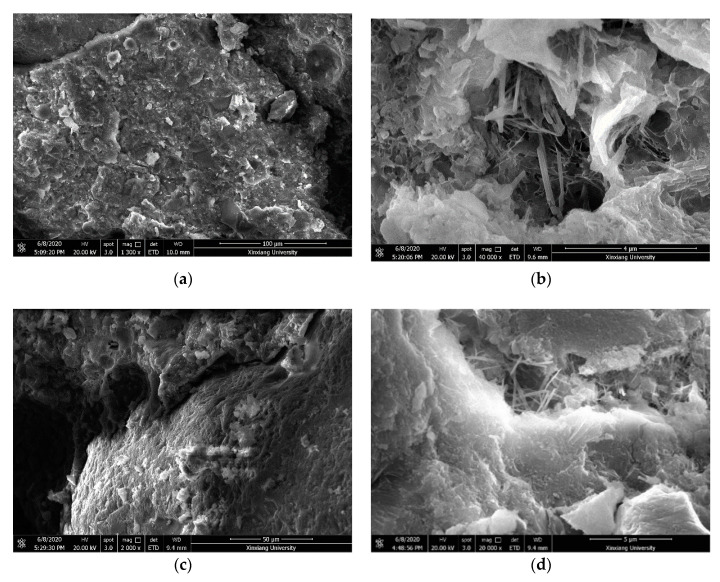
Microstructure of (**a**) Control, (**b**) NSC-0.5, (**c**) MRC-5, (**d**) MR5N3.

Figure 1 shows images from SEM taken on samples of concrete. The hydration product of the control concrete is flake crystal, with large pores and loose structure; the large pores are due to the insufficient hydration of the cement, resulting in a large number of C–H crystals in the specimen shown in Figure 1a; at the same time, the number of flocculated and reticulated C–S–H and rod-shaped aft produced by the hydration reaction is relatively small, and the resulting structure is not dense. After adding a certain amount of nano-SiO_2_, the compactness of the cement concrete is significantly improved, and the pore content is also significantly reduced, which indicates that nano-SiO_2_ improves the internal structure of cement concrete. The reason is that the size of the nano-SiO_2_ particles is small. According to the surface and small size effect, the number of atoms on the surface increases. Under the conditions of more unsaturated bonds, higher surface energy and chemical activity of particles, it is easy to combine with other atoms to form a uniform and stable state. The addition of silylated rubber can change the microstructure of concrete particles and pores, and improve the adhesion between the rubber particles and the concrete [23]. The addition of silanated rubber and nano-SiO_2_ has an effect on the microstructure of concrete. There is basically no crack on the boundary between hydration products and concrete aggregates, and they are closely linked to each other. The distribution of hydration products is particularly uniform. The C–S–H and AFt of the structure are more than ettringite AFt. No large pores and voids were found in the microstructure.

### 3.2. Mass Loss and Relative Dynamic Modulus of Elasticity

**Figure 2:**
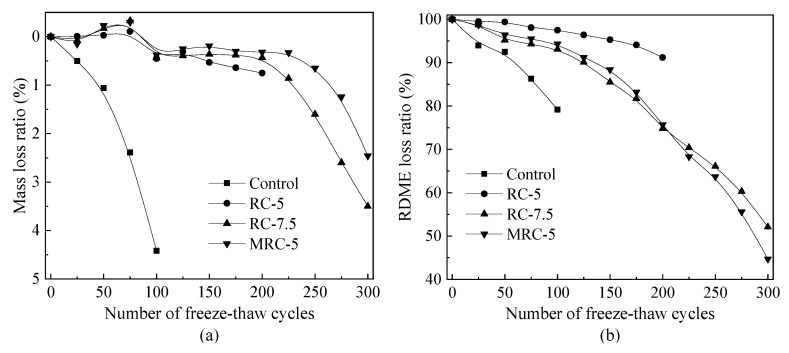
Loss ratios of concrete with rubber (**a**) Mass, (**b**) RDME.

The frost resistance of concrete is usually described and characterized by mass loss and the relative dynamic modulus of elasticity (RDME) [24]. Figure 2 presents the loss ratios of mass and RDME of the designed concretes with rubber aggregates. Generally, the reference concrete (control mixture) has poor frost resistance, which shows a very large mass and RDME loss ratios even after 100 freeze–thaw cycles, namely 4.42% and 68.1%, respectively. After adding 5% or 7.5% rubber aggregates, all concrete mixtures show longer freeze–thaw lives. Their mass loss ratios do not have an obvious decrease even when subjected to 200 freeze–thaw cycles. However, both mass and RDME have a sharp reduction beyond 200 freeze–thaw cycles. Compared to RC-5, RC-7.5 shows just slightly less mass loss, which indicates that too high a rubber content is not suggested in frost-resistant concrete. Concrete with 5% silanized rubber aggregates (MRC-5) has less mass loss than the RC-5 mixture, which is in line with the positive effect on compressive strength in Section 3.1. 

**Figure 3:**
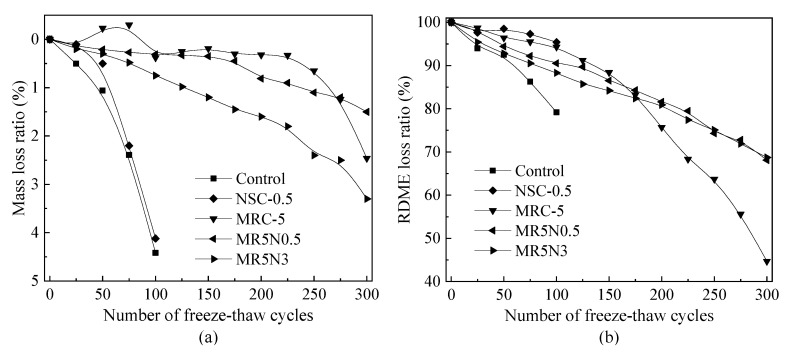
Loss ratios of concrete with rubber and nano-SiO_2_ (**a**) Mass, (**b**) RDME.

Figure 3 shows the loss ratios of mass and RDME of the designed concretes incorporated with both rubber and nano-SiO_2_. In the presence of nano-SiO_2_, the frost resistance of concrete is enhanced based on the results of the mass loss and RDME loss. However, the improvement on mass stability is not as much as the RDME. Frost-resistant concrete incorporated with both silanized rubber and nano-SiO_2_ can retain relatively higher mass and RDME values even suffering from 300 freeze–thaw cycles. For example, the MR5N0.5 and MR5N3 still have approximately 70% RDME. It indicates that there exits a positively synergistic effect between silanized rubber and nano-SiO_2_ on frost resistance.

In this study, the mass of the concrete firstly increases because of absorbing the water. After more than 125 freeze–thaw cycles, the mass of the specimen declines. This phenomenon also fully shows that when evaluating the frost resistance of concrete, its mass loss is a variable parameter. When the parameter changes to a negative, it indicates that the concrete has a macro failure [25]. The compressive strength and elastic modulus decrease linearly with the increase in freeze–thaw cycles, especially after 250 freeze–thaw cycles. However, the change trend of RDME is quite different. After 50 freeze–thaw cycles, the RDME begins to decrease sharply, down to around 15% reduction at 175 freeze–thaw cycles. Qin et al. [26] studied the microscopic changes of the pore structure of hardened concrete with freeze–thaw cycles. The results show that the concrete with 0.405 mm pore spacing coefficient and 2.38% air content can still withstand more than 300 freeze–thaw cycles. The severe microdamage occurs after about 200 freeze–thaw cycles, and then freeze–thaw damage increases rapidly.

To sum up, it is necessary to add appropriate contents of the rubber aggregate and nano-SiO_2_ for developing frost-resistant concretes, considering both mechanical strength and freeze–thaw resistance. This study recommends using 5% silanized rubber and 3% nano-SiO_2_, namely, a mixture of MR5N3.

### 3.3. Chord Length Distribution of Pores

**Figure 4:**
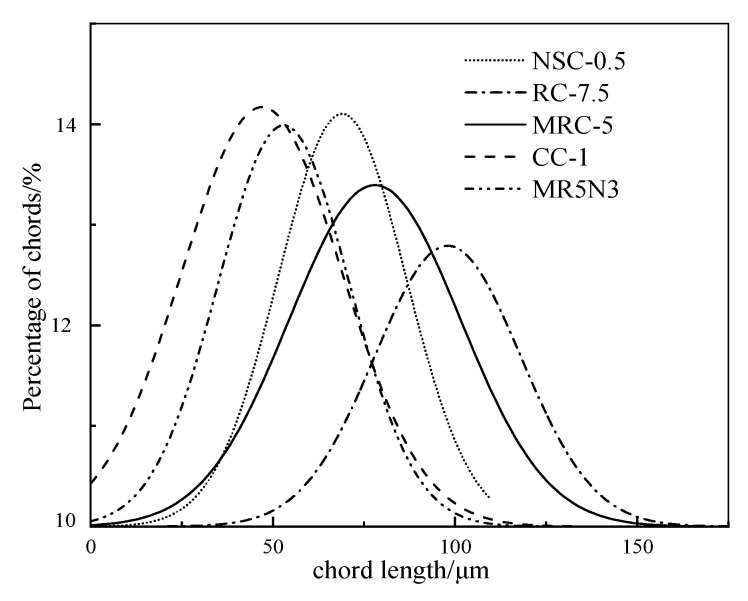
Chord length distribution of pores.

Figure 4 shows the pore chord length distributions of the designed concretes. The chord length to the peak presents an order as RC-7.5 > MRC-5 > NSC-0.5 > MR5N3 > Control. Thus, the reference concrete mixture has more small pores than the other mixtures. The rubber and nano-SiO_2_ tend to decrease the total pores. The addition of nano-SiO_2_ could density the microstructure by the pozzolanic effect. It helps to improve the hydration degree and produce more C–S–H gel by reacting with Ca(OH)_2_, and then fills the small pores [27]. The concretes with rubber aggregates have similar broad peaks to the reference, but the chord length to the peak tends to larger, which means they have more of the larger pores. Leonid et al. [28] investigated the development of the pore size distribution after freeze–thaw cycles by using a mercury intrusion porosimeter. The increased porosity during freeze–thaw damage usually concentrates in small and medium pores from 25 nm to 150 nm. The critical pore dimeter tends to become larger and larger with the increase in freeze–thaw cycles.

**Figure 5:**
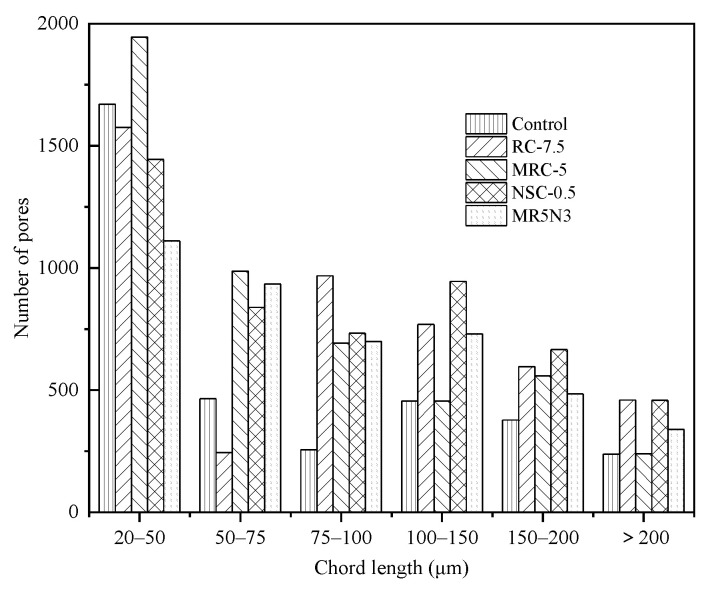
Number vs. chord length.

**Figure 6:**
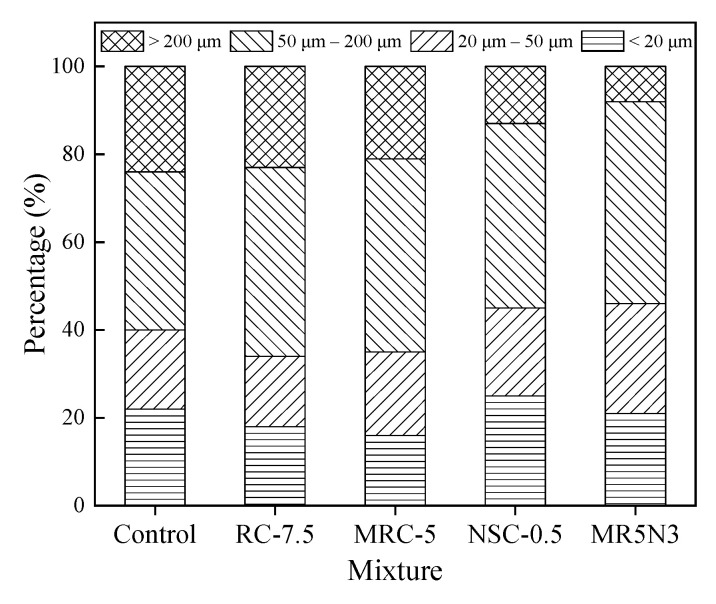
Percentages of pore types.

Figure 5 and Figure 6 show the numbers and proportions of the different pore ranges, respectively. The pore size smaller than 200 μm occupies more than 75% of the total pores, and the medium pores (20 μm–50 μm) make up a larger part than the other pore types. The pore structures of the frost-resistant concretes incorporated with rubber and nano-SiO_2_ are greatly enhanced. The addition of rubber aggregates is the most important factor in the pore structure, after which are the silanized rubber aggregates, and rubber and nano-SiO_2_. In this study, the mixture of MR5N3 has the appropriate contents of silanized rubber and nano-SiO_2_, occupying more than 90% of pores smaller than 200 μm, which results in its improved properties such as compressive strength and frost resistance.

### 3.4. Specific Surface Area of Pores 

**Figure 7:**
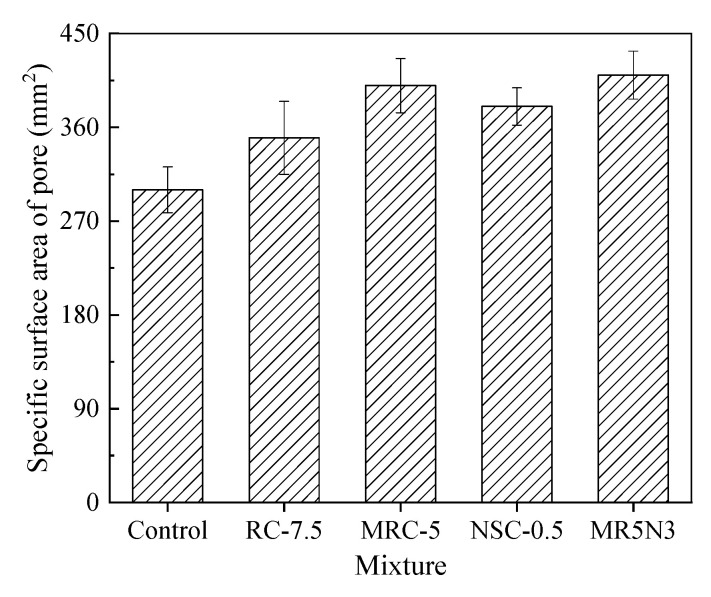
Specific surface area of pores.

**Figure 8:**
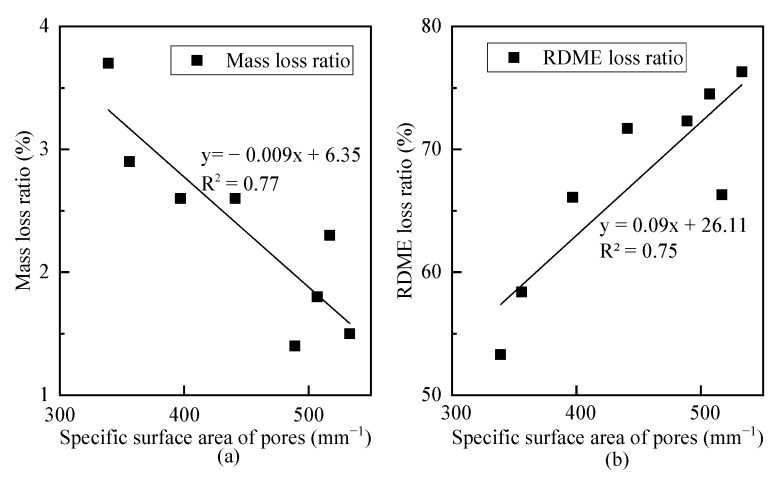
Correlations between pore specific surface area and loss ratio (**a**) mass, (**b**) RDME.

Figure 7 shows the specific surface area of pores. Concretes incorporated with rubber or/and nano-SiO_2_ have a relatively higher specific surface area of pores, which is in line with the research by Zhu et al. [29]. To understand the effect of pore specific surface area on frost resistance, the correlations between pore specific surface area and mass loss and RDME loss are analyzed, as illustrated in Figure 8. Two linear models are proposed to describe their relationships. With the increase in pore specific surface area, the frost resistance of concrete increases with a lower mass loss ratio and a higher RDME ratio.

**Figure 9:**
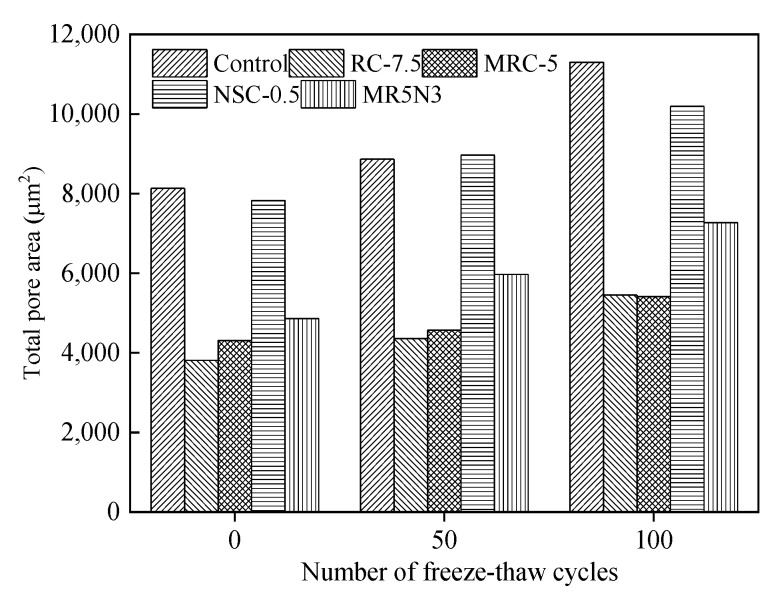
Influence of freeze–thaw cycles on total pore area.

Figure 9 presents the influence of freeze–thaw cycles on the total pore area. After a certain number of freeze–thaw cycles, the total pore areas of the concretes tend to be increased. Compared with the modified frost-resistant concrete, the total pore area in the reference concrete has the largest initial value and the fastest development. The increase in total pore area is reduced by utilizing normal or silanized rubber aggregates. The concrete with nano-SiO_2_ shares a similar total pore area to that in the control concrete. It indicates that rubber can strengthen the elasticity of pores, and then improve the frost resistance. 

### 3.5. Air Content

**Figure 10:**
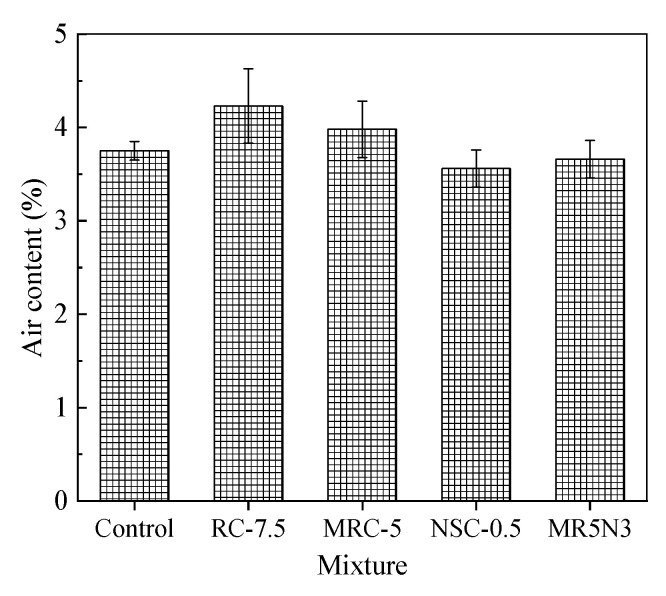
Air content of concrete.

**Figure 11:**
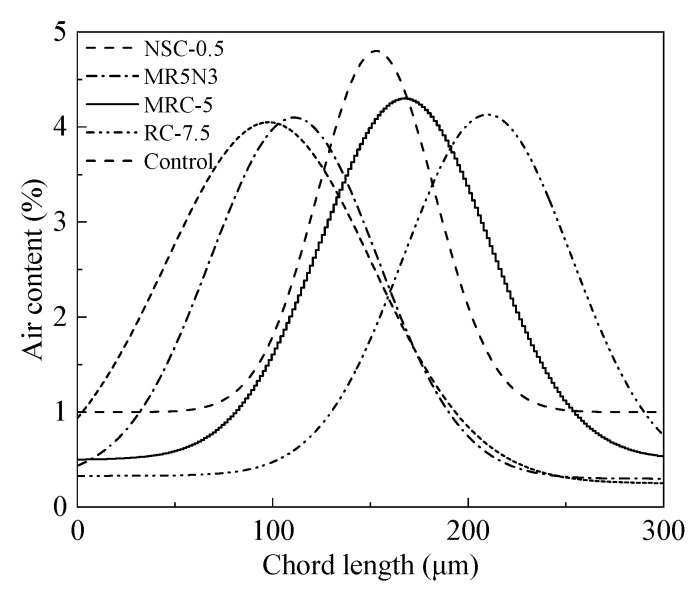
Relationship between chord length distribution and air content.

**Figure 12:**
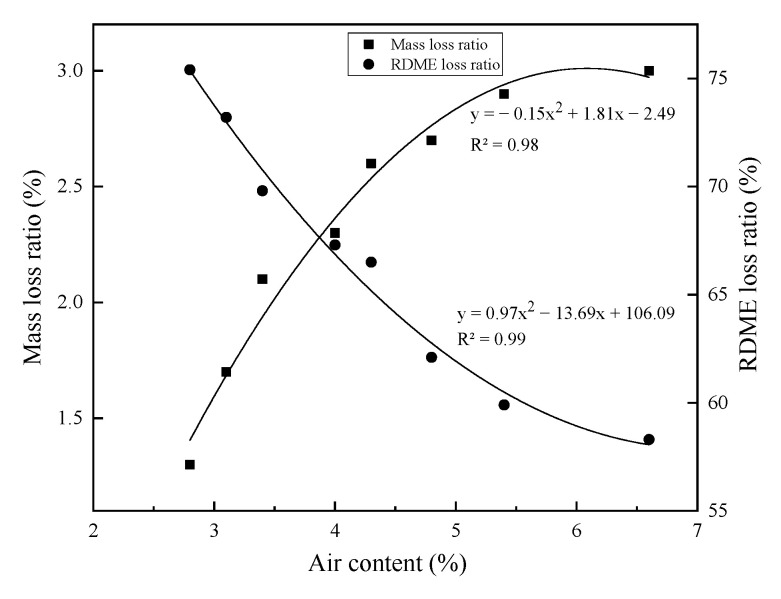
Relationship between air content and frost resistance.

The air content or porosity greatly affects the frost resistance of the hardened concrete. Figure 10 shows the air contents in different concretes. The addition of rubber aggregates results in increased air contents, while nano-SiO_2_ contributes to slightly reduced air contents. The air content distributions vary greatly for the different concrete mixtures, as shown in Figure 11. The air content presents an order as RC-7.5 > MRC-5 > NSC-0.5 > MR5N3 > Control. The air content distribution in Figure 11 shows a similar tendency to the chord length distribution in Figure 4. This study also researches the effect of the air content on the mass loss and RDME of the concretes, as shown in Figure 12. With the increase in air content in concrete, the mass loss after the freeze–thaw test is reduced and RDME is kept at a relatively high percentage. Hence, a high air content usually leads to a better frost resistance. The mass loss can be reduced by 1.5% and RDME is increased by 10% with an additional air content of 1%. The slopes of the fitting curves become smaller in the conditions of air content beyond 4%. Therefore, frost-resistant concrete incorporated with rubber and nano-SiO_2_ is suggested at less 4% air content. 

**Figure 13:**
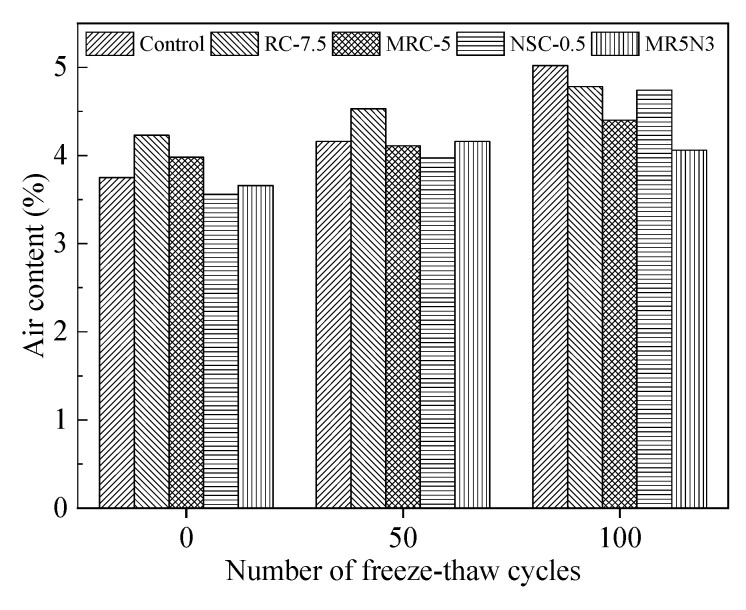
Influence of freeze–thaw cycle on air content.

To analyze the change of air content during freeze–thaw damage, the air content development is tested after each of 50 times freeze–thaw cycles, as shown in Figure 13. The air contents show continuous increase with the increase in freeze–thaw cycles. The influence of the freeze–thaw cycles on air content development in different concretes is very similar. After suffering from 50 times freeze–thaw cycles, all air contents appear just slightly increased, while an obvious increase can be observed after 100 times freeze–thaw cycles. The reference concrete without any rubber and nano-SiO_2_ shows the fastest development of air content, which means its freeze–thaw damage propagates rapidly. The mixture of MR5N3 has the slowest air content development, which indicates 5% silanized rubber aggregates and 3% nano-SiO_2_ have positive synergistic effect on frost resistance. The fine rubber aggregates can fill in some pores and especially improve the elasticity of pores, then enhance the frost resistance [30]. However, the interfacial bond between the normal rubber and the matrix is very weak, because of none chemical reaction between the normal rubber and the cementitious matrix. Thus, the modified rubber aggregates after silanization are suggested to increase the interfacial bond and then frost resistance. 

**Figure 14:**
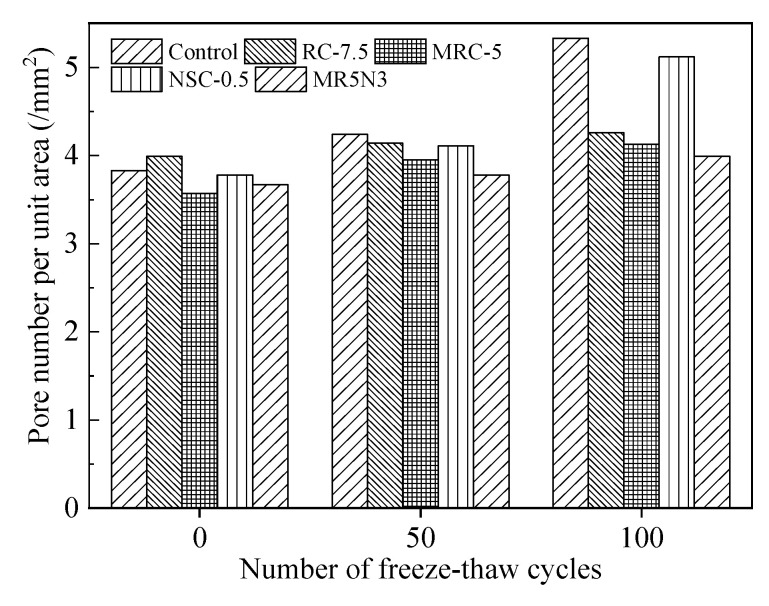
Influence of freeze–thaw cycle on pore number per unit area.

Meanwhile, this paper also conducts statistical analysis on the pore number per unit area in concrete after freeze–thaw cycles. A higher increased pore number per unit area indicates a worse frost resistance, as shown in Figure 14 and Table 6. The freeze–thaw damage occurs due to the combined action of repeated frost expansion stress and continuous water migration [31]. The time-dependent change of pores reflects the freeze–thaw damage mechanism of the concrete. The freeze–thaw action first occurs around the large pores, leading to microcrack damage, and water moves into the interior of concrete along the microcrack. This results in the increased number of macropores at initial stage of freeze–thaw cycles. With the increase in freeze–thaw cycles, the water continues to migrate, which causes more minor damage under repeated freeze–thaw stress, and then the number of small pores increases significantly. After long-term repeated freeze–thaw cycles, the small and medium pores are interconnected, the number of large pores in the structure increases sharply, and the concrete is damaged due to deterioration.

With the increase in freeze–thaw cycles, the absolute number of pores increases but the growth rates decrease. Based on analysis of the pore number growth rate after different freeze–thaw cycles, it shows that the pore number increases sharply in the first 50 freeze–thaw cycles, especially in the middle pore. The pore size changes from large to small in the order of Control > NSC0.5 > RC-7.5 > MRC-5 > MR5N3, and the growth rate of extra large pores is the smallest. At the beginning of the freeze–thaw cycle, the micro and nanopores expand under the action of frost, and microdamage occurs, which is manifested by the sharp increase in the number of small and medium pores in the concrete. With the gradual increase in freeze–thaw times, the microdamage leads to the interconnection and expansion of small pores, forming macropores, and the number of pores at all levels increases with the increase in freeze–thaw times. The increase in the number of extra large pores is the main factor affecting the freeze–thaw damage.

### 3.6. Average Pore Spacing Coefficient

**Figure 15:**
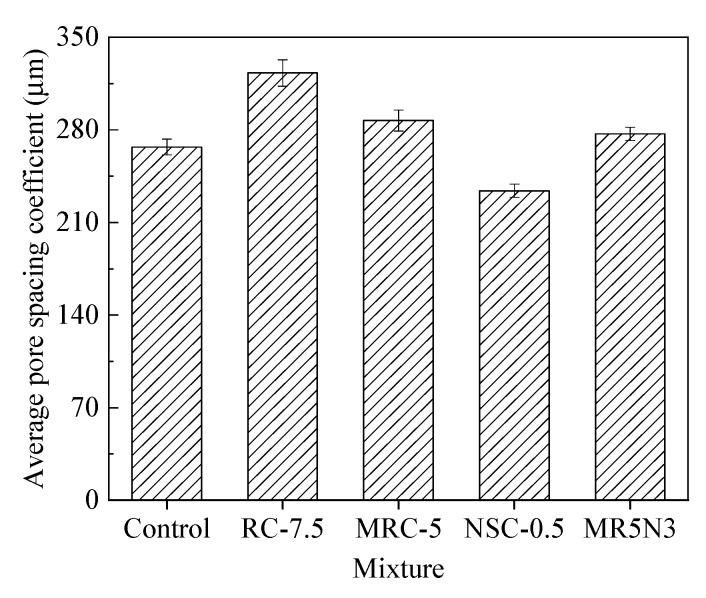
Average pore spacing coefficient of concrete.

The pore spacing coefficient represents the average distance between each pore, which is the most important factor affecting the frost resistance of concrete. A larger pore spacing coefficient results in a worse frost resistance, due to the greater hydrostatic pressure and osmotic pressure produced by water in concrete pores during freezing and thawing. On the contrary, if the pore spacing coefficient is smaller, the frost resistance of concrete is better. The pore spacing coefficients of the different concretes are shown in Figure 15. The average pore spacing coefficient from small to large is NSC-0.5 < Control < MR5N3 < MRC-5 < RC-7.5.

**Figure 16:**
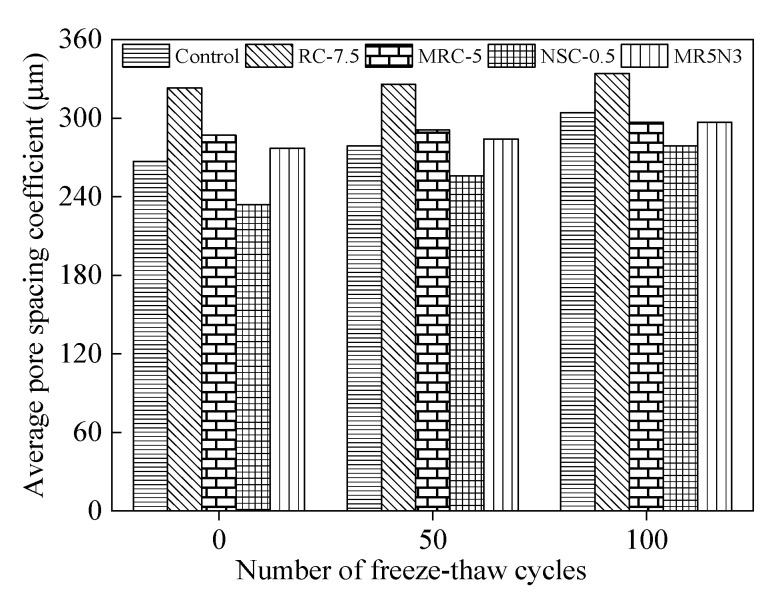
Influence of freeze–thaw cycle on average pore spacing coefficient.

Figure 16 analyzes the development of the pore spacing coefficient after freeze–thaw cycles. According to the Fagerlund theory [32], the hydrostatic pressure caused by water freezing in concrete pores is directly proportional to the square of the pore spacing. A larger pore spacing leads to a longer flow of liquid water into adjacent pores. The more time it takes, the greater the water pressure generated by the water flowing through capillary channels. When the water pressure exceeds the compressive strength of the concrete, the concrete failure occurs. Generally, an air entraining agent is used in concrete to introduce closed and relatively uniform micropores, then alleviate the expansion of the water pressure of pores and cut off the water seepage channel, thus improving the frost resistance of the concrete [33].

**Figure 17:**
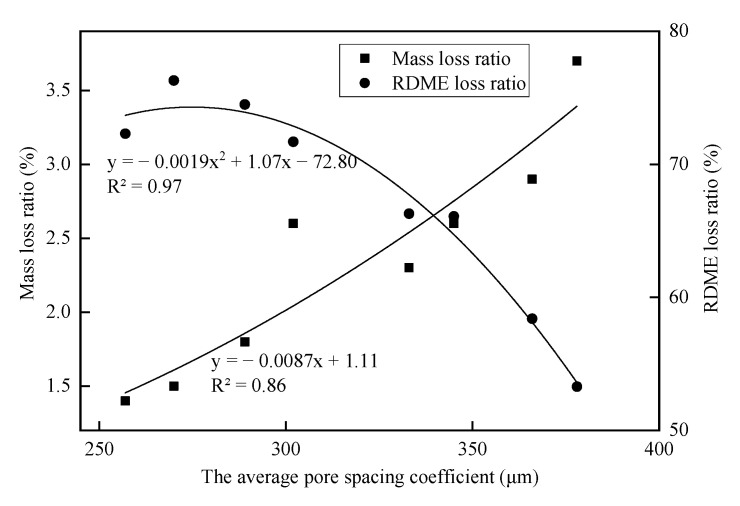
Relationship between average pore spacing coefficient and frost resistance.

This paper also researches the relationship between average pore spacing coefficient *L* and frost resistance including mass loss and RDME, as illustrated in Figure 17. With the increase in the pore spacing coefficient, the mass loss rate increases and the RDME decreases: namely, the frost resistance of the concrete decreases. After 300 freeze–thaw cycles, the RDME of the concrete with silanized rubber and nano-SiO_2_ is more than 65%, while the mass loss rate is less than 4%. The best frost-resistant concrete corresponds to the pore spacing coefficient of about 330 μm. Therefore, the frost resistance of the concrete incorporated with silanized rubber and nano-SiO_2_ is better than that of ordinary concrete.

## 4. Conclusions

This paper develops frost-resistant concretes incorporated with rubber aggregates and nano-SiO_2_, and investigates their pore structures and freeze–thaw damage mechanism. The mass loss, relative dynamic modulus of the elastic, chord lengths, specific surface areas, contents and spacing coefficients of pores are tested. The main conclusions of in this paper can be summarized:

- The addition of an appropriate content of rubber aggregates could greatly improve the frost resistance of concrete, but usually decreases the mechanical strength. It is necessary to add nano-SiO_2_ to partially compensate for the strength loss. The silanization of the rubber could further enhance the interfacial bond between the rubber and the matrix, thus improving strength and frost resistance.
- Considering both compressive strength and frost resistance, concrete incorporated with 5% silanized rubber and 3% nano-SiO_2_ is the best modified mixture, which is recommended for developing frost-resistant concrete.
- The damage degree develops with the increase in freeze–thaw cycles, resulting in the continuous reduction of mass loss and the dynamic modulus of elasticity. The highly elastic rubber improves the stiffness, pore size and distribution, which thus significantly increase frost resistance.
- A more homogenous pore distribution can withstand a higher expansion pressure generated by freezing, which contributes to a better frost resistance. With the increase in freeze–thaw cycles, both pore area and diameter increase, and the increased degree of the pore diameter is more remarkable than that of the area.
- The pore structure and frost resistance can be characterized by the chord length, specific surface area, air content and average pore spacing coefficient. This research proposes to comprehensively utilize those parameters as quantitative evaluation indexes of frost resistance.

The main conclusions have verified that silanized rubber and nano-SiO_2_ can improve the frost resistance of concrete, which will become an effective tool for improving the performance of concrete. Further research is necessary to investigate the effects of the long-term performance of the frost-resistant concrete and its practical engineering applications.

## Figures and Tables

**Table 1:** Physical properties and chemical composition of used cement.

Substance	CaO	SiO_2_	Al_2_O_3_	Fe_2_O_3_	MgO	SO_2_
%	64.14	20.45	4.36	2.98	1.07	2.26
Specific surface area (m^2^/kg)			331			
Specific density (g/cm^3^)			3.15			
Initial setting time (min)			149			
Final setting time (min)			204			

**Table 2:** Properties of used nano-SiO_2_.

Appearance	Average Diameter (nm)	Specific Surface Area (m^2^/kg)	LOI (%)	Composition
White powder	≤20	586	4.36	SiO_2_ ≥ 99.9

**Table 3:** Recipe of control concrete (kg/m^3^).

Mixture	Cement	Fly Ash	Sand	Aggregate	Water	Plasticizer
Control	461	51	586	1087	215	0.4

**Table 4:** Research parameters of designed frost-resistant concretes.

Mixture	Nano-SiO_2_ (by the Mass of Powder, %)	Rubber (by the Mass of Cement, %)	Silanized Rubber (by the Mass of Cement, %)	44.4
NSC-0.5	0.5%	--	--	41.6
RC-5	--	5%	--	35.7
RC-7.5	--	7.5%	--	33.5
MRC-5	--	--	5%	37.3
MR5N0.5	0.5%	--	5%	30.6
MR5N3	3%	--	5%	38.4

**Table 5:** Pore types in concrete.

Pore Size	Extra Large Pore	Large Pore	Medium Pore	Small Pore
Diameter (µm)	>200	50–200	20–50	<20

**Table 6:** Increased rate of pore numbers under freeze–thaw cycles.

Mixture	Freeze–Thaw Cycles	Increased Rate of Pore Number (%)			
		Small Pore <20 μm	Medium Pore 20–50 μm	Large Pore 50–200 μm	Extra Large Pore >200 μm
Control	0–50	36.1	35.5	23.3	3.6
	50–100	19.5	26.5	27.1	2.2
RC-7.5	0–50	18.7	21.5	22.2	0.5
	50–100	10.4	12.2	15.8	0.2
MRC-5	0–50	17.5	19.6	18.4	0.1
	50–100	9.6	11.2	13.2	0.3
NSC-0.5	0–50	34.1	32.0	23.6	2.9
	50–100	15.3	14.6	18.5	2.1
MR5N3	0–50	16.4	16.8	15.6	2.0
	50–100	13.6	14.7	15.1	1.1

## Data Availability

Data is contained within the article.

## References

[1] Sharafutdinov E., Shon C.-S., Zhang D., Chung C.-W., Kim J., Bagitova S. (2019). Frost resistance number to assess freeze and thaw resistance of non-autoclaved aerated concretes containing ground granulated blast-furnace slag and micro-silica. Materials.

[2] Fu Y., Cai L., Yonggen W. (2011). Freeze–thaw cycle test and damage mechanics models of alkali-activated slag concrete. Constr. Build. Mater..

[3] Guo B.L. (2013). Relational analysis of air voids characteristic parameters of hardened concrete and freezing-thawing durability. J. Highw. Transp. Res. Dev..

[4] Hasholt M.T., Christensen K.U., Pade C. (2019). Frost resistance of concrete with high contents of fly ash—A study on how hollow fly ash particles distort the air void analysis. Cem. Concr. Res..

[5] Fuyuan G., Koichi M. (2018). Multi-scale simulation of freeze-thaw damage to RC column and its restoring force characteristics. Eng. Struct..

[6] Powers T.C. (1949). The air requirement of frost resistant concrete. Proc. Highw. Res. Board.

[7] Wang Z., Zeng Q., Wu Y., Wang L., Yao Y., Li K. (2014). Relative humidity and deterioration of concrete under freeze-thaw load. Constr. Build. Mater..

[8] Kim K.Y., Yun T.S., Choo J., Kang D.H., Shin H.S. (2012). Determination of air-void parameters of hardened cement-based materials using X-ray computed tomography. Constr. Build. Mater..

[9] Cheng Y., Huang F., Qi S., Li W., Liu R., Li G. (2020). Durability of concrete incorporated with siliceous iron tailings. Constr. Build. Mater..

[10] Zarauskas L., Skripkiūnas G., Girskas G. (2017). Influence of Aggregate Granulometry on Air Content in Concrete Mixture and Freezing*—*Thawing Resistance of Concrete. Procedia Eng..

[11] Zhang R., Liu P., Ma L., Yang Z., Chen H., Zhu H.X., Xiao H., Li J. (2020). Research on the Corrosion/Permeability/Frost Resistance of Concrete by Experimental and Microscopic Mechanisms Under Different Water–Binder Ratios. Int. J. Concr. Struct. Mater..

[12] Ahin Y., Akkaya Y., Tademir M.A. (2020). Effects of freezing conditions on the frost resistance and microstructure of concrete. Constr. Build. Mater..

[13] Zhao Y.-R., Wang L., Lei Z.-K., Han X.-F., Shi J.-N. (2018). Study on bending damage and failure of basalt fiber reinforced concrete under freeze-thaw cycles. Constr. Build. Mater..

[14] Hasholt M.T. (2014). Air void structure and frost resistance: A challenge to Powers’ spacing factor. Mater. Struct..

[15] Xu J.-H., Feng X.-T., Chen S.-L. (2012). Effects of rubber aggregate on the frost resistance of concrete. Dongbei Daxue Xuebao J. Northeast. Univ..

[16] Zhu T.K. (2015). Research on the Microstructure and Performance of Rubber Cement Mortar.

[17] Duan P., Shui Z., Chen W., Shen C. (2013). Effects of metakaolin, silica fume and slag on pore structure, interfacial transition zone and compressive strength of concrete. Constr. Build. Mater..

[18] Tang J., Zou C., Xue H., Niu D., Zheng X., Zhao Q., Jun W. (2019). Study on pore evolution characteristics of double-doped molded bag concrete. Bull. Chin. Ceram. Soc..

[19] Qin X.-C., Meng S.-P., Cao D.-F., Tu Y.-M., Sabourova N., Grip N., Ohlsson U., Blanksvärd T., Sas G., Elfgren L. (2016). Evaluation of freeze-thaw damage on concrete material and prestressed concrete specimens. Constr. Build. Mater..

[20] GB/T50082-2009 Standard Method for Long Term Performance and Durability Test of Ordinary Concrete. https://max.book118.com/html/2019/0608/7130160052002032.shtm.

[21] Jarolím T., Brožovský J., Šácha D., Pizúrová N. (2018). Influence of CNT Addition to the Frost Resistance of Concrete. Key Eng. Mater..

[22] Leonid D. (2019). Design estimation of concrete frost resistance. Constr. Build. Mater..

[23] Shen Y., Liu J., Zhou S., Li G. (2019). Experimental investigation on the freeze–thaw durability of concrete under compressive load and with joints. Constr. Build. Mater..

[24] Federowicz K., Figueiredo V.A., Al-kroom H., Abdel-Gawwad H.A., Abd Elrahman M., Sikora P. (2020). The Effects of Temperature Curing on the Strength Development, Transport Properties, and Freeze-Thaw Resistance of Blast Furnace Slag Cement Mortars Modified with Nanosilica. Materials.

[25] Xu J.Y., Li Z.C., Luo X. (2014). Comparative Study on the frost-resisting properties of rubber powder concrete. Bull. Chin. Ceram. Soc..

[26] Zhong J., Shi J., Shen J., Zhou G., Wang Z. (2019). Investigation on the Failure Behavior of Engineered Cementitious Composites under Freeze-Thaw Cycles. Materials.

[27] Li J., Han X.Y. (2015). Effect of Microstructure Characteristics of Concrete on Durability. J. Lanzhou Inst. Technol..

[28] Krstic M., Davalos J.F., Rossi E., Figueiredo S.C., Copuroglu O. (2021). Freeze-Thaw Resistance and Air-Void Analysis of Concrete with Recycled Glass–Pozzolan Using X-ray Micro-Tomography. Materials.

[29] Gorzelańczyk T., Schabowicz K. (2019). Effect of Freeze-Thaw Cycling on the Failure of Fibre-Cement Boards, Assessed Using Acoustic Emission Method and Artificial Neural Network. Materials.

[30] Wolter S., Uhre F.A.H., Hasholt M.T., Dahl V.A., Anton F. (2019). Air void analysis of hardened concrete by means of photogrammetry. Constr. Build. Mater..

[31] Eriksson D., Wahlbom D., Malm R., Fridh K. (2021). Hygro-thermo-mechanical modeling of partially saturated air-entrained concrete containing dissolved salt and exposed to freeze-thaw cycles. Cem. Concr. Res..

[32] Gou Y., Zhang L., Liu C., Zhang H., Wei C., Cai X., Yang H., Guan Q., Zhai S., Liu L. (2021). Investigation of freeze-thaw mechanism for crumb rubber concrete by the online strain sensor. Measurement.

[33] Rosenqvist M., Pham L.-W., Terzic A., Fridh K., Hassanzadeh M. (2017). Effects of interactions between leaching, frost action and abrasion on the surface deterioration of concrete. Constr. Build. Mater..

